# Establishing a cancer driver gene signature-based risk model for predicting the prognoses of gastric cancer patients

**DOI:** 10.18632/aging.203948

**Published:** 2022-03-14

**Authors:** Jun Chen, Chao Zhou, Ying Liu

**Affiliations:** 1 Department of Oncology, The First Affiliated Hospital of Nanchang University, Nanchang 330006, Jiangxi, People's Republic of China; 2 Department of Neurology, Jiangxi Provincial People's Hospital Affiliated to Nanchang University, Nanchang 330006, Jiangxi, People's Republic of China; 3 Department of Emergency, The First Affiliated Hospital of Nanchang University, Nanchang 330006, Jiangxi, People's Republic of China

**Keywords:** gastric cancer, cancer driver gene, risk signature, nomogram, prognosis

## Abstract

Despite the high prevalence of gastric cancer (GC), molecular biomarkers that can reliably detect GC are yet to be discovered. The present study aimed to establish a robust gene signature based on cancer driver genes (CDGs) that can predict GC prognosis. Transcriptional profiles and clinical data from GC patients were analyzed using univariate Cox regression analysis and the least absolute shrinkage and selection (LASSO)-penalized Cox regression analysis to select optimal prognosis-related genes for modeling. Time-dependent receiver operating characteristic (ROC) and Kaplan-Meier analyses were done to assess the predictive power of this gene signature. A nomogram model for prediction of survival of GC patients was established using the CDG signature and clinical information, and a seven-CDG signature was identified. Risk scores were calculated using this signature, and patients were subsequently divided into high- and low-risk groups; high-risk patients in the training and validation datasets had poorer prognoses than low-risk patients. Cox regression analysis revealed that the CDG signature is an independent prognostic factor for GC. The signature and other clinical features were used to construct a nomogram for predicting overall GC patient survival. Calibration and decision curve analysis showed that the nomogram accurately predicted survival, highlighting its clinical utility. Thus, we established a novel CDG signature and nomogram for predicting GC prognosis, which may facilitate personalized treatment of GC.

## INTRODUCTION

Gastric cancer (GC) is one of the most common forms of gastrointestinal cancer and is associated with very high morbidity and mortality rates [1]. It can be histologically classified into various subtypes, including adenocarcinoma, squamous cell carcinoma, adenosquamous carcinoma, and carcinoid. Gastric adenocarcinoma accounts for 80-90% of all GC cases. In recent years, the incidence of GC has increased and GC cases have been associated with poor prognoses. Currently, surgical resection is the main option for GC treatment [2]. Therefore, identifying new therapeutic targets for GC is required [3]. Over the past few decades, several studies that focused on developing molecular targeted therapies for GC and understanding their underlying molecular mechanisms have shed light on GC pathogenesis [4]. However, despite the importance of accurate classification and risk stratification of GC patients in improving management decisions and prognosis predictions, reliable biomarkers to predict GC prognosis are lacking [5, 6].

Cancers are complicated diseases characterized by uncontrolled cellular growth, invasion, and metastasis, which are primarily caused by genetic mutations [7, 8]. These mutations are termed “drivers” due to their ability to drive tumorigenesis and confer certain selective advantages to somatic tissue cells over their neighboring cells. Mutations in cancer driver genes (CDGs) affect cellular homeostasis and numerous cellular processes. Recently, Francisco et al. reported molecular-level changes that occur during malignant tumor progression [7]. Their study represents the most comprehensive analysis performed to date, as they analyzed over 28,000 samples of 66 cancer types and revealed 568 CDGs; these results suggest the involvement of a variety of molecular mechanisms. CDGs are important factors that affect the occurrence and development of GC, and they play important roles in GC prognosis. This necessitates the development of a robust and reliable CDG signature to improve individualized survival predictions for GC.

This study aimed to build a scoring model by classifying GC patients on the basis of a CDG signature, in combination with other clinicopathological factors, to improve the ability to predict GC patient prognoses, thereby helping to guide individualized treatment. This study represents the first investigation into the clinical value of CDGs in predicting GC prognoses. CDGs are expected to become novel key GC biomarkers and open new avenues for the development of novel GC treatment methods.

## RESULTS

### Construction of the CDG signature for GC

Overlapping prognostic CDGs from The Cancer Genome Atlas Stomach Adenocarcinoma (TCGA-STAD) and GSE62254 databases were selected to determine the candidate CDGs. Twenty-four CDGs were identified for final analysis (Supplementary Figure 1A). Next, least absolute shrinkage and selection operator (LASSO)-penalized Cox analysis was performed to narrow down the list of CDGs, and 12 genes were identified for downstream analyses (Supplementary Figure 1B). Multivariate Cox analysis was performed based on the CDGs selected by LASSO analysis (Supplementary Figure 1C). The prognostic risk scores of the CDG signature were determined as follows:

Risk score = (-0.06570) * (expression level of Damage Specific Deoxyribose Nucleic Acid [DNA] Binding Protein 2 [DDB2]) + 0.04589 * (expression level of Aminopeptidase [ENPEP]) + 0.00243* (expression level of Guanine Nucleotide binding protein, Alpha Stimulating activity polypeptide [GNAS]) - 0.10790 * (expression level of Musashi Ribose Nucleic Acid [RNA] Binding Protein 2 [MSI2]) + 0.14947 * (expression level of myosin Va [MYO5A]) + 0.22932 * (expression level of Pleomorphic Adenoma Gene 1 [PLAG1]) - 0.18526 * (expression level of RNA Binding Motif 15 [RBM15]; Supplementary Figure 1D).

### CDG expression and its mutations in GC

**Figure 1:**
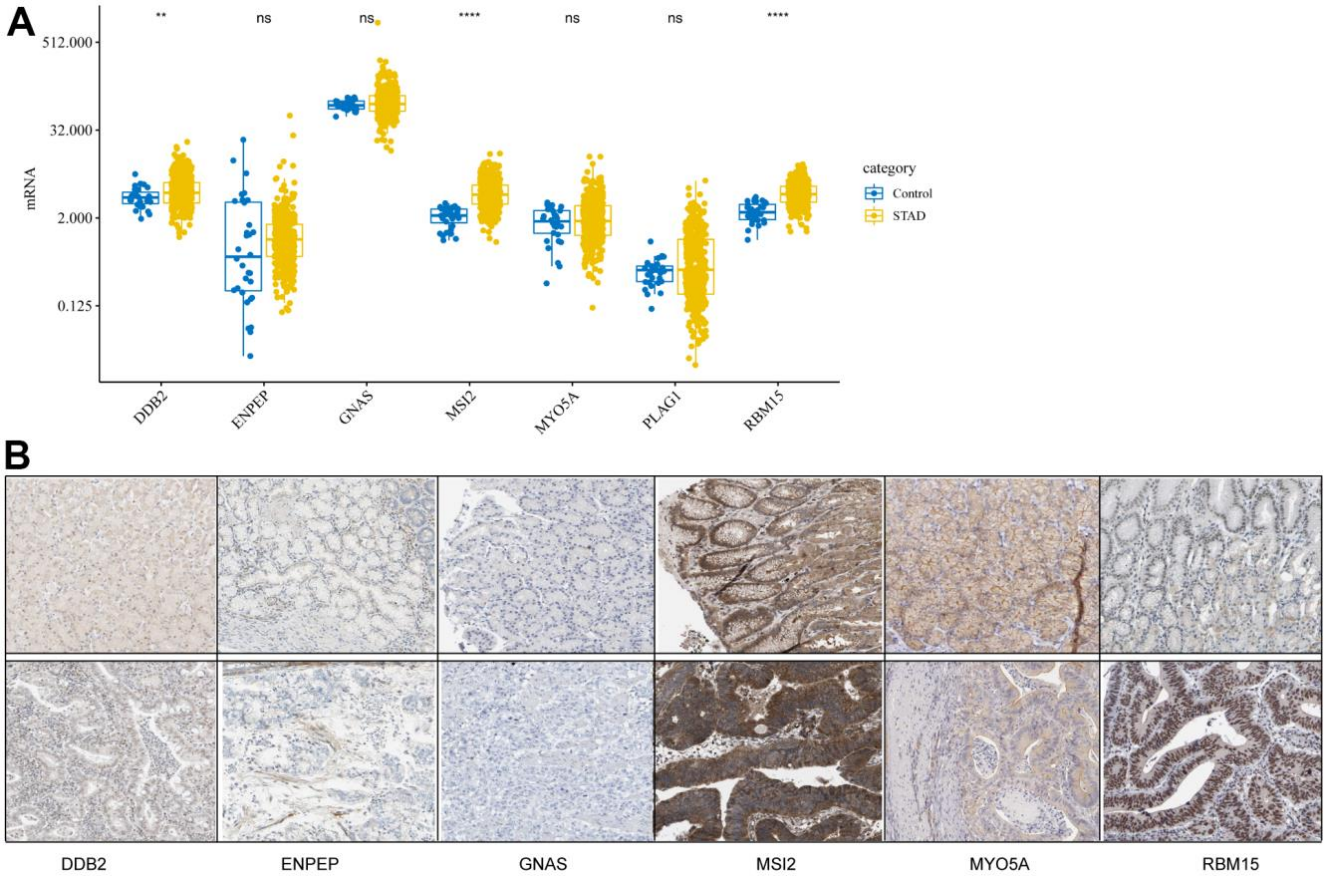
**Expression levels of cancer driver genes (CDGs) and their alterations in gastric cancer (GC).** (**A**) CDG mRNA expression levels in GC obtained from The Cancer Genome Atlas Stomach Adenocarcinoma (TCGA-STAD). (**B**) Expression levels of proteins encoded by CDGs in normal tissues as obtained from the Human Protein Atlas (HPA) database (Data for *GLAP1* was not available at HPA database).

To study the differences in CDG expression between tumor and normal tissues, we examined CDG messenger RNA (mRNA) levels in samples from TCGA-STAD. The results revealed that tumor tissues had significantly higher expression of *DDB2*, *MSI2*, and *RBM15* than the normal tissues (Figure 1A). However, no differences were observed in the expression of *ENPEP*, *GNAS*, *MYO5A*, or *PLAG1*. We also examined the levels of proteins encoded by these CDGs using clinical samples from the Human Protein Atlas (HPA) database and found that the differences in protein levels were consistent with the observed differences in mRNA levels (Figure 1B).

### The prognostic value of the CDG signature

**Figure 2:**
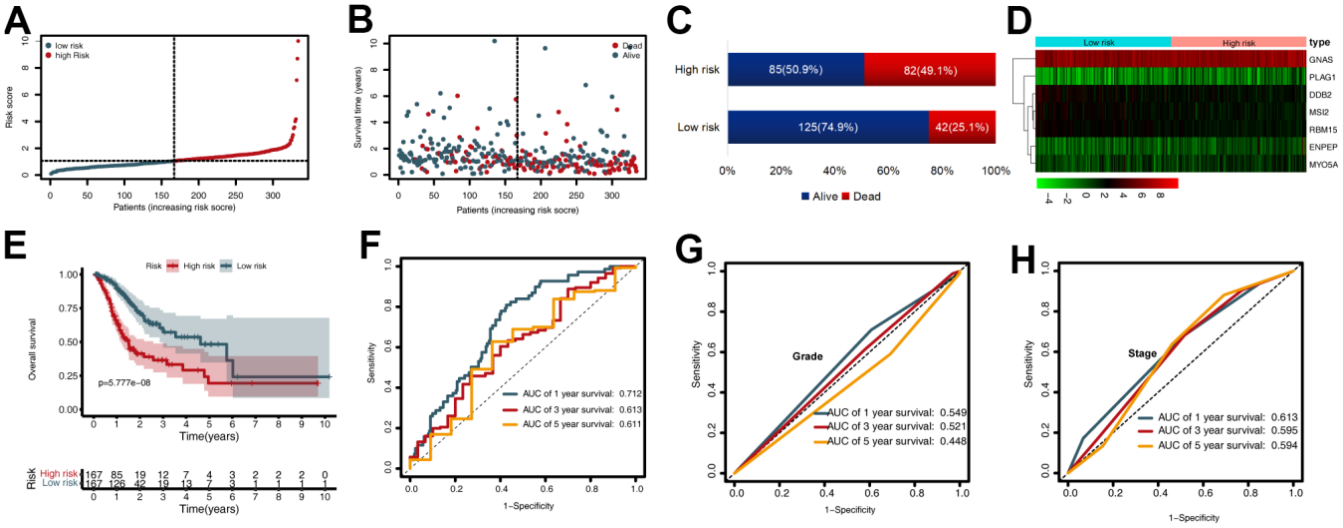
**Prognostic value of the cancer driver gene (CDG) signature in The Cancer Genome Atlas (TCGA) training set.** (**A**) Distribution of risk scores per patient. (**B**) Relationships between overall survival (OS) status and survival time of gastric cancer (GC) patients ranked on the basis of risk score. (**C**) Comparison of mortality risk between the two groups in TCGA cohort. (**D**) Heatmap representing the expression profiles of the seven CDGs. (**E**) Kaplan-Meier analysis of OS between high- and low-risk groups in TCGA set. (**F**) Time-dependent receiver operating characteristic (ROC) analysis for OS prediction in TCGA set. (**G**) Time-dependent ROC analysis for grade prediction in TCGA set of OS. (**H**) Time-dependent ROC analysis for stage prediction in TCGA set of OS.

To evaluate the prognostic value of the CDG signature in the training set, the GC patients were divided into high-risk (*n* = 167) and low-risk (*n*= 167) groups according to the median risk score. The corresponding signature risk score survival statuses were ranked and displayed on a dot-plot (Figure 2A, 2B). Individuals exhibited a greater risk of mortality with increasing risk score (Figure 2C). Heatmaps of the seven prognostic CDGs are displayed in Figure 2D.

**Figure 3:**
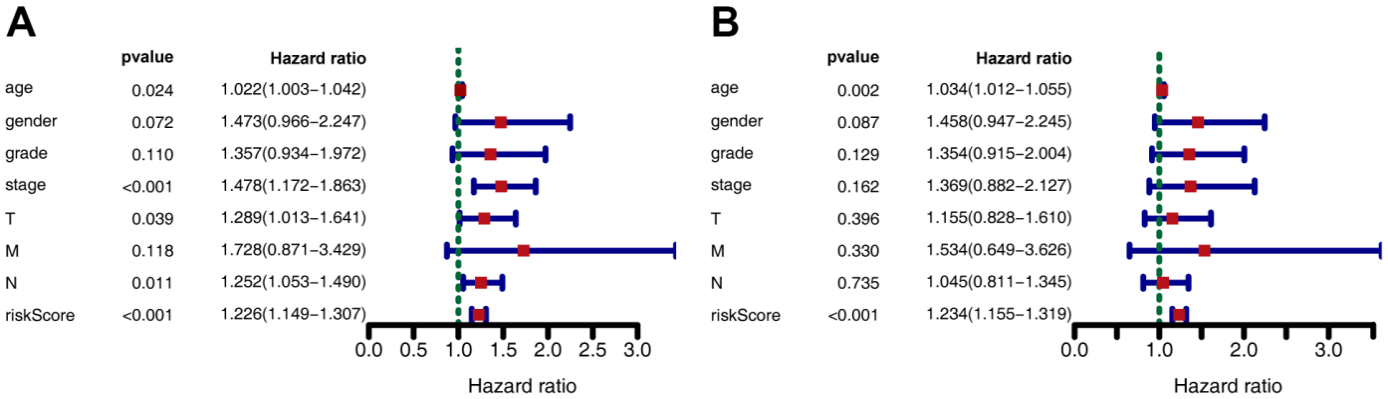
**Forest plot depicting associations between risk factors and other clinical features and the prognosis of gastric cancer.** (**A**) Univariate Cox regression analysis. (**B**) Multiple Cox regression analysis.

Kaplan-Meier analysis revealed that in the training set, patients in the high-risk group had shorter overall survival (OS) than those in the low-risk group (*P* < 0.001) (Figure 2E). Time-dependent receiver operating characteristic (ROC) analysis demonstrated that the area under the curve (AUC) values for one year, three years, and five years were 0.712, 0.613, and 0.611, respectively (Figure 2F). We also compared the prognostic value to traditional clinicopathological predictors. Time-dependent ROC analyses for grade-based and stage-based prediction of OS (TCGA) are shown in Figure 2G, 2H. Univariate and multivariate Cox regression analyses showed that age and risk score were both independent prognostic factors for GC (Figure 3A, 3B).

### Validation of the CDG signature

**Figure 4:**
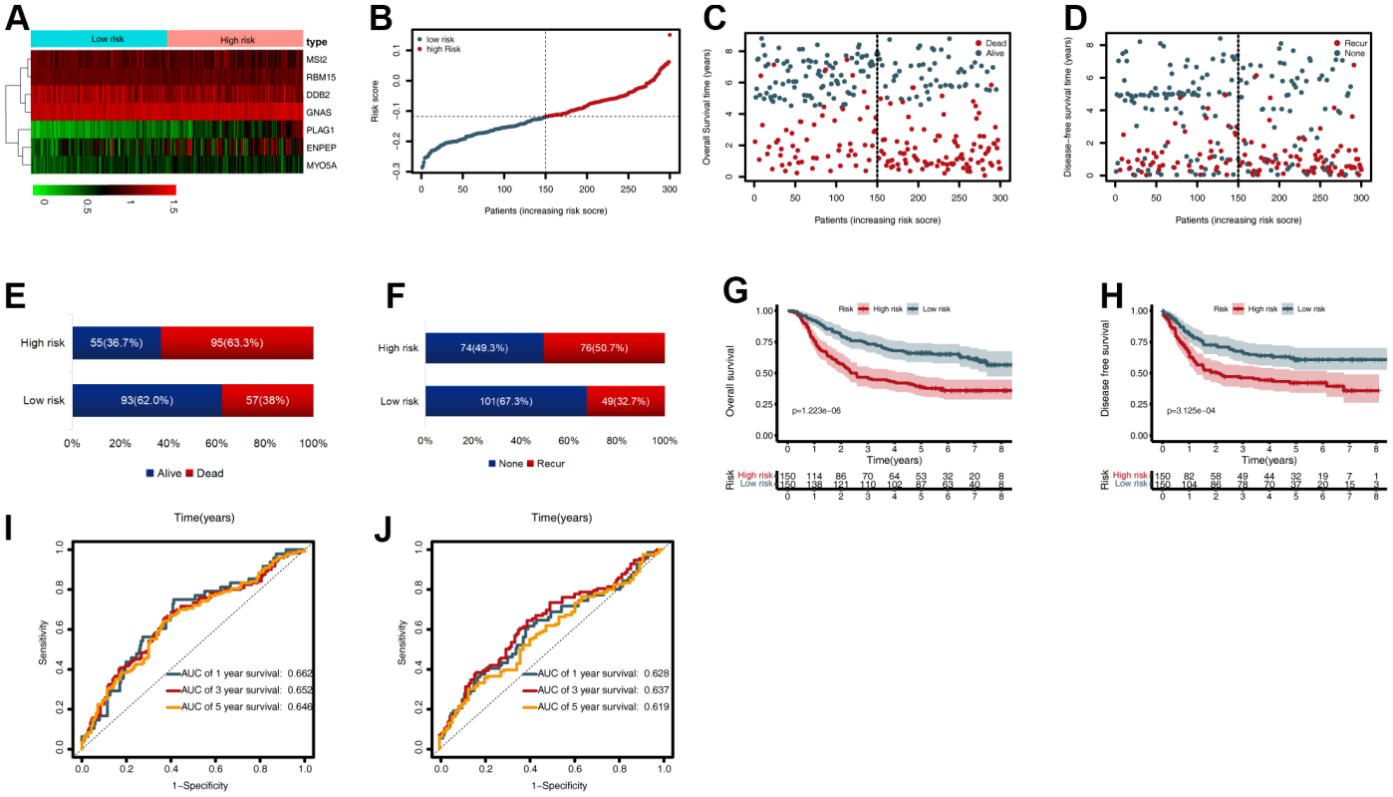
**External validation of the cancer driver gene (CDG) signature using the gastric cancer (GC) data from the Gene Expression Omnibus (GEO) validation set.** (**A**) Heatmap representing expression profiles of the seven CDGs. (**B**) Distribution of risk scores per patient. (**C**) Relationships between overall survival (OS) status and survival time in GC patients ranked by risk score. (**D**) Relationships between disease-free survival (DFS) status and survival time in GC patients ranked by risk score. (**E**) Comparison of OS risk between the two groups. (**F**) Comparison of DFS risk between the two groups. (**G**) Kaplan-Meier analysis of OS between high- and low-risk groups in GSE62254. (**H**) Kaplan-Meier analysis of DFS between high- and low-risk groups in GSE62254. (**I**) Time-dependent receiver operating characteristic (ROC) analysis for OS prediction in the GSE62254 cohort. (**J**) Time-dependent ROC analysis for DFS prediction in the GSE62254 cohort.

To validate the CDG signature, patients in the validation set were divided into high- and low-risk groups, according to the median risk score (calculated using the CDG signature). The results were compatible with those obtained in the training set derived from TCGA. Figure 4A shows the heatmap of the seven prognostic CDGs. The corresponding signature risk score survival statuses were ranked and are displayed as a dot-plot (Figure 4A–4C). Individuals were at greater risk of mortality and recurrence with increasing risk scores (Figure 4E, 4F). Kaplan-Meier analysis also showed that the high-risk group had shorter OS and disease-free survival (DFS) than the low-risk group (*P* < 0.001; Figure 4G, 4H). Time-dependent ROC analysis of OS showed that the 1-, 3-, and 5-year AUC values were 0.662, 0.652, and 0.646 respectively (Figure 4I). The AUC values of one-, three-, and five-year DFS were 0.628, 0.637, and 0.619, respectively (Figure 4J).

### Subgroup analysis of the CDG signature

**Figure 5:**
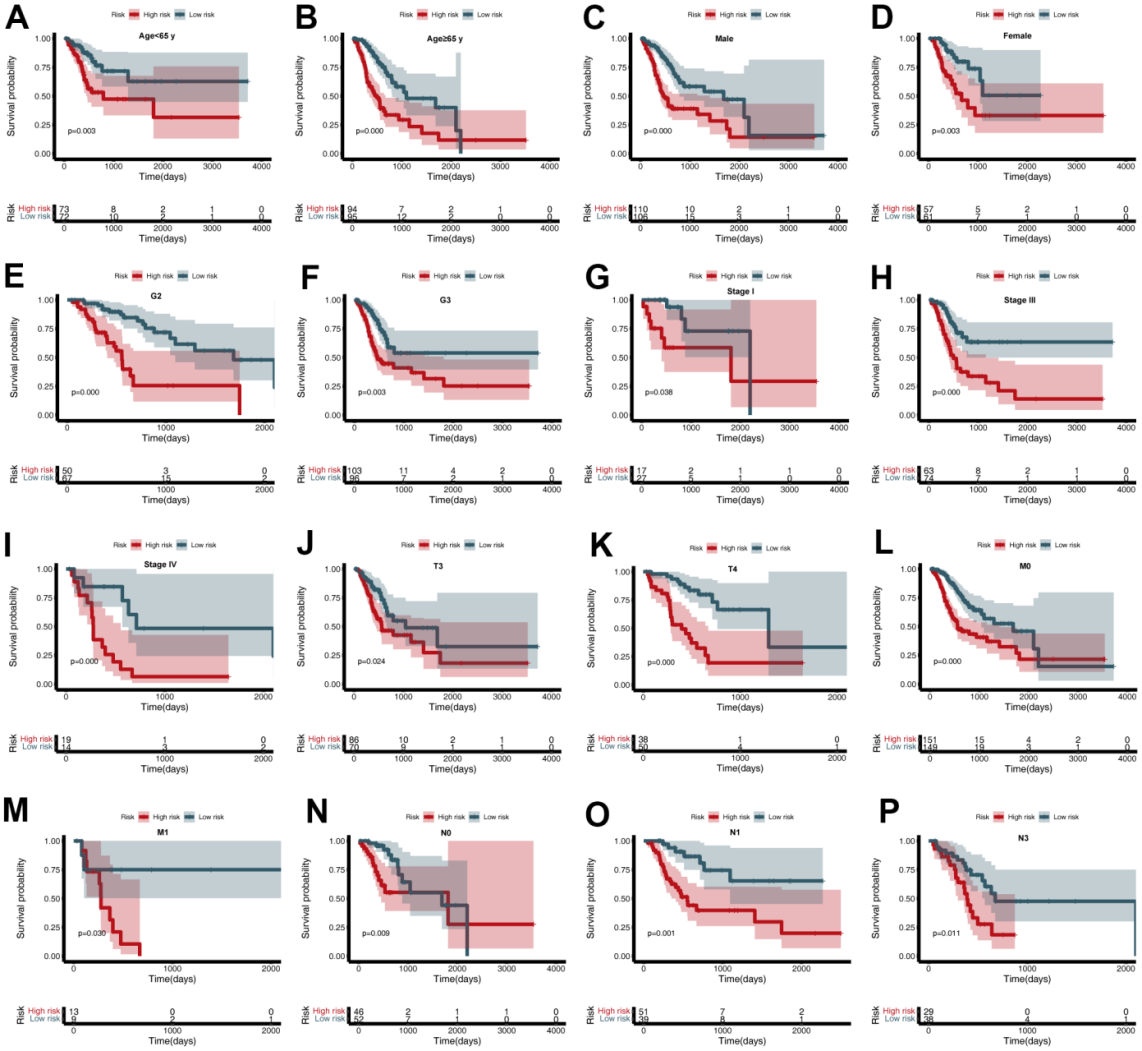
**Subgroup analysis of the cancer driver gene (CDG) signature.** (**A**) Age < 65 years, (**B**) Age ≥ 65 years, (**C**) Male, (**D**) Female, (**E**) G2, (**F**) G3, (**G**) Stage I, (**H**) Stage III, (**I**) Stage IV, (**J**) T3, (**K**) T4, (**L**) M0, (**M**) M1, (**N**) N0, (**O**) N1, and (**P**) N3.

To further estimate the utility of the CDG signature in predicting survival outcomes, stratification analysis was conducted based on specific clinicopathological characteristics. These subgroups included age (<65 or ≥65 years), gender, grade, stage, and T, N, and M stages (Figure 5A–5P). Stratification of the training and validation datasets revealed that the CDG signature could categorize patients into different survival groups and provide statistically significant prognostic values (Tables 1, 2).

**Table 1:** Stratified survival analyses based on clinical characteristics and CDG signature in TCGA-STAD cohort.

**Characteristics**	**Number**		**%**	**Overall survival**	
	**High-risk**	**Low-risk**		**HR (95% CI)**	**P value**
**Age (years)**					
< 65	73	72	43.4%	2.627(1.378-5.007)	0.003
≥ 65	94	95	56.6%	2.791(1.761-4.424)	0.000
**Sex**					
Male	110	106	64.7%	2.623(1.682-4.090)	0.000
Female	57	61	35.3%	1.705(1.204-2.415)	0.003
**Grade**					
G1	8	1	2.7%	-	-
G2	50	67	35.0%	2.115(1.518-2.947)	0.000
G3	103	96	59.6%	1.554(1.219-1.981)	0.000
Unknown	6	3	2.7%	-	-
**Stage**					
I	17	27	13.2%	1.940(1.037-3.629)	0.038
II	58	48	31.7%	1.225(0.844-1.777)	0.286
III	63	74	41.0%	1.717(1.290-2.284)	0.000
IV	19	14	9.9%	2.111(1.251-3.563)	0.005
Unknown	10	4	4.2%	-	-
**T stage**					
T1	2	12	4.2%	-	-
T2	37	35	21.6%	1.425(0.944-2.150)	0.092
T3	86	70	46.7%	1.359(1.041-1.774)	0.024
T4	38	50	26.3%	2.228(1.539-3.225)	0.000
Unknown	4	0	1.2%	-	-
**M stage**					
M0	151	149	89.8%	1.576(1.290-1.925)	0.000
M1	13	9	6.6%	2.360(1.087-5.122)	0.030
Unknown	3	9	3.6%	-	-
**N stage**					
N0	46	52	29.3%	1.708(1.146-2.544)	0.009
N1	51	39	26.9%	2.044(1.346-3.105)	0.001
N2	32	36	20.4%	1.467(0.974-2.210)	0.067
N3	29	38	20.1%	1.587(1.113-2.263)	0.011
Unknown	9	2	3.3%	-	-

HR, hazard ratio; CI, confidence interval.

**Table 2:** Stratified survival analyses based on clinical characteristics and CDG signature in the GSE62254 cohort.

**Characteristics**	**Number**		**%**	**Overall survival**		**Disease-free survival**	
	**High-risk**	**Low-risk**		**HR (95% CI)**	***P* value**	**HR (95% CI)**	***P* value**
**Age (years)**							
< 65	81	80	53.7%	2.431(1.491-3.963)	0.000	2.201(1.318-3.675)	0.003
≥ 65	69	70	46.3%	1.443(1.154-1.805)	0.001	1.299(1.007-1.675)	0.044
**Sex**							
Male	103	96	66.3%	1.260(1.032-1.538)	0.023	1.155(0.928-1.436)	0.197
Female	47	54	33.7%	2.074(1.538-2.796)	0.000	2.019(1.454-2.805)	0.000
**Lauren pathological classification**							
Intestinal	59	87	48.7%	1.450(1.124-1.869)	0.004	1.327(1.003-1.757)	0.048
Diffuse	82	53	45.0%	1.476(1.157-1.884)	0.002	1.388(1.068-1.805)	0.014
Mixed	9	10	6.3%	1.251(0.722-2.170)	0.425	1.062(0.548-2.058)	0.859
**Stage**							
I	7	23	10.0%	0.937(0.313-2.805)	0.908	1.272(0.383-4.227)	0.694
II	41	56	32.3%	1.638(1.140-2.352)	0.008	1.156(0.760-1.760)	0.498
III	56	40	32.0%	1.341(1.008-1.784)	0.044	1.278(0.932-1.754)	0.128
IV	46	31	25.7%	1.263(0.975-1.637)	0.077	1.228(0.932-1.619)	0.145
**T stage**							
T1+T2	75	113	62.7%	1.510(1.202-1.896)	0.000	1.370(1.056-1.777)	0.018
T3+T4	75	37	37.3%	1.217(0.953-1.556)	0.116	1.091(0.843-1.411)	0.509
**M Stage**							
M0	132	141	91.0%	1.441(1.205-1.722)	0.000	1.348(1.109-1.638)	0.003
M1	18	9	9.0%	4.708(1.538-14.411)	0.007	2.186(0.702-6.803)	0.177
**N Stage**							
N0	12	26	12.7%	1.123(0.561-2.246)	0.744	1.301(0.615-2.752)	0.491
N1+N2+N3	138	124	87.3%	1.473(1.240-1.749)	0.000	1.345(1.116-1.620)	0.002

HR, hazard ratio; CI, confidence interval.

### Gene set enrichment analysis (GSEA)

**Figure 6:**
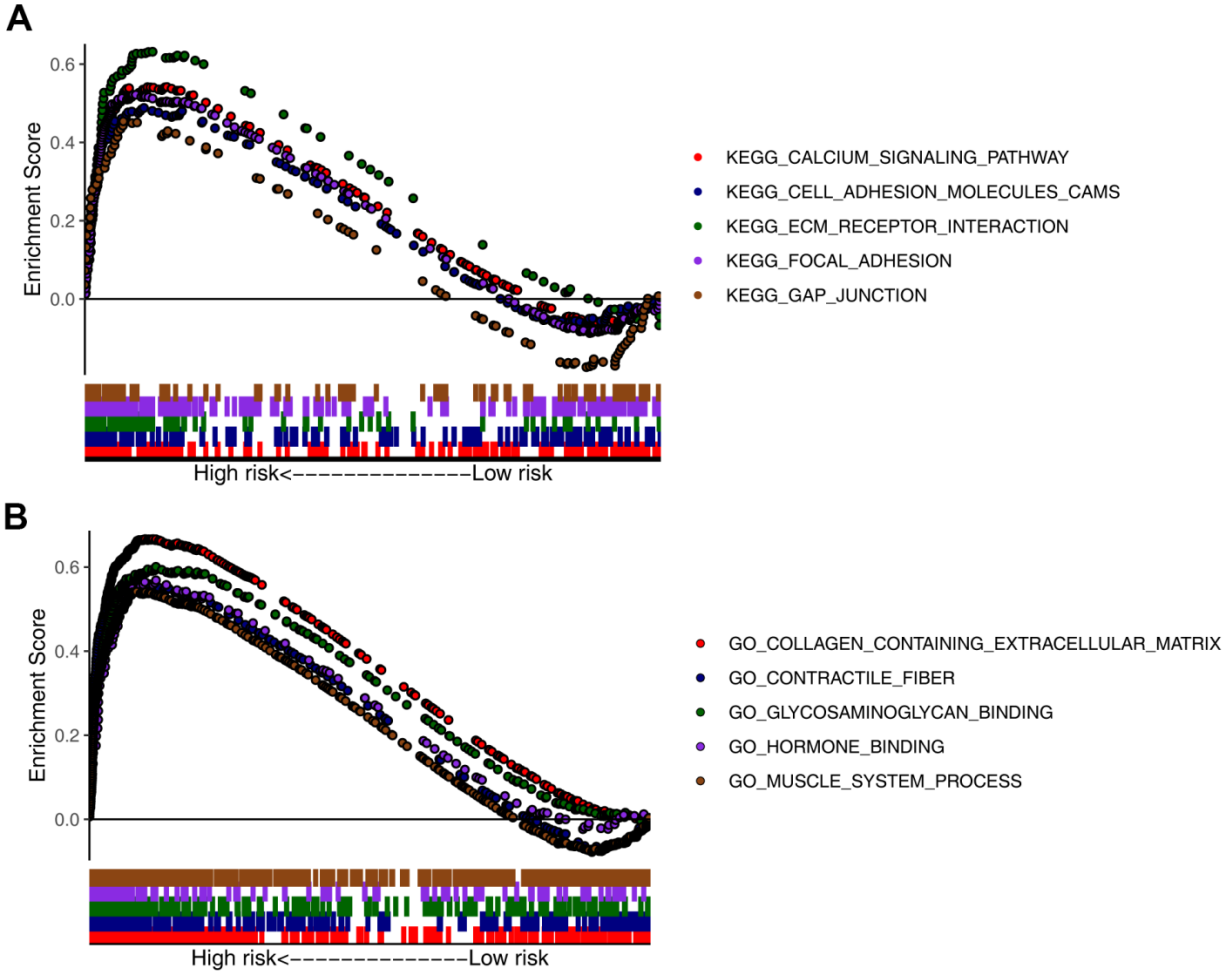
**Gene set enrichment analysis (GSEA) of high- and low-risk groups.** Top five representatives from (**A**) Gene Ontology (GO) term enrichment analysis and (**B**) Kyoto Encyclopedia of Genes and Genomes (KEGG) pathway enrichment analysis.

To further analyze the functions of the seven CDGs identified, GSEA was conducted for the high- and low-risk patients in the four datasets. GSEA results for the Kyoto Encyclopedia of Genes and Genomes (KEGG) pathways indicated that the “calcium signaling pathway,” “cell adhesion molecules,” (CAMs) “extracellular matrix receptor interaction,” “focal adhesion,” and “gap junction” categories were highly enriched in the high-risk group (Figure 6A). GSEA results for Gene Ontology (GO) terms indicated that the “collagen-containing extracellular matrix,” “contractile fiber,” “glycosaminoglycan binding,” “hormone binding,” and “muscle system processes” were highly enriched in the high-risk group (Figure 6B).

### Analysis of tumor immunity

**Figure 7:**
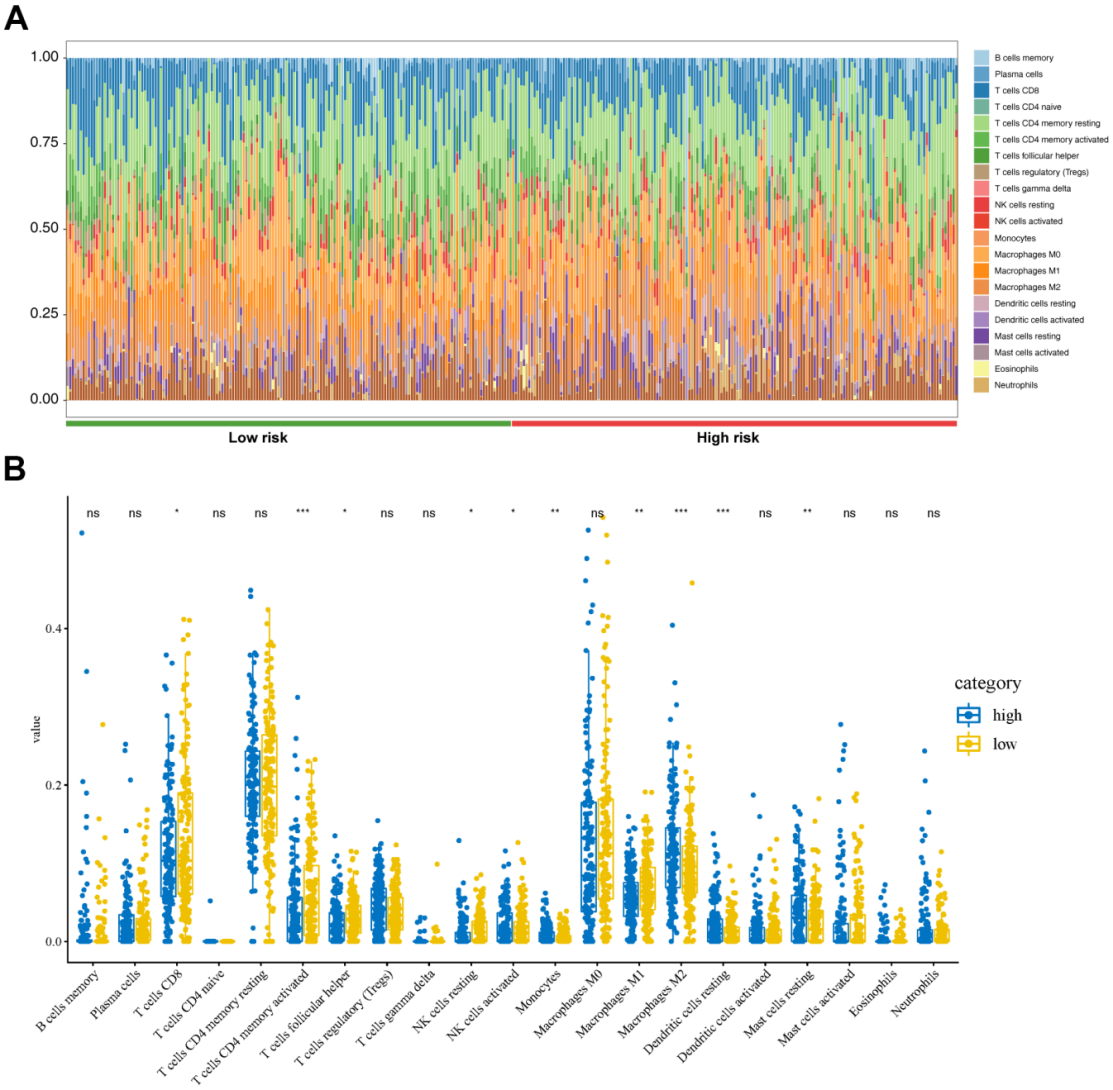
**Tumor immunity analysis based on the CDG signature.** (**A**) Relative proportion of immune cells between high- and low-risk groups. (**B**) Violin plot depicting differences in the abundances of 22 types of immune cells between the high- and low-risk groups.

TCGA-STAD gene expression matrix was uploaded to the Cell type Identification by Estimating Relative Subsets of RNA Transcripts (CIBERSORT) platform to estimate the proportions of the 22 immune cell types. The high-risk group had high proportions of activated natural killer (NK) cells, monocytes, M2 macrophages, resting dendritic cells, and resting mast cells (Figure 7A). The low-risk group had high proportions of CD8 T-cells, CD4 memory-activated T-cells, follicular helper T-cells, resting NK cells, and M1 macrophages (Figure 7B).

### Establishment and evaluation of the nomogram

**Figure 8:**
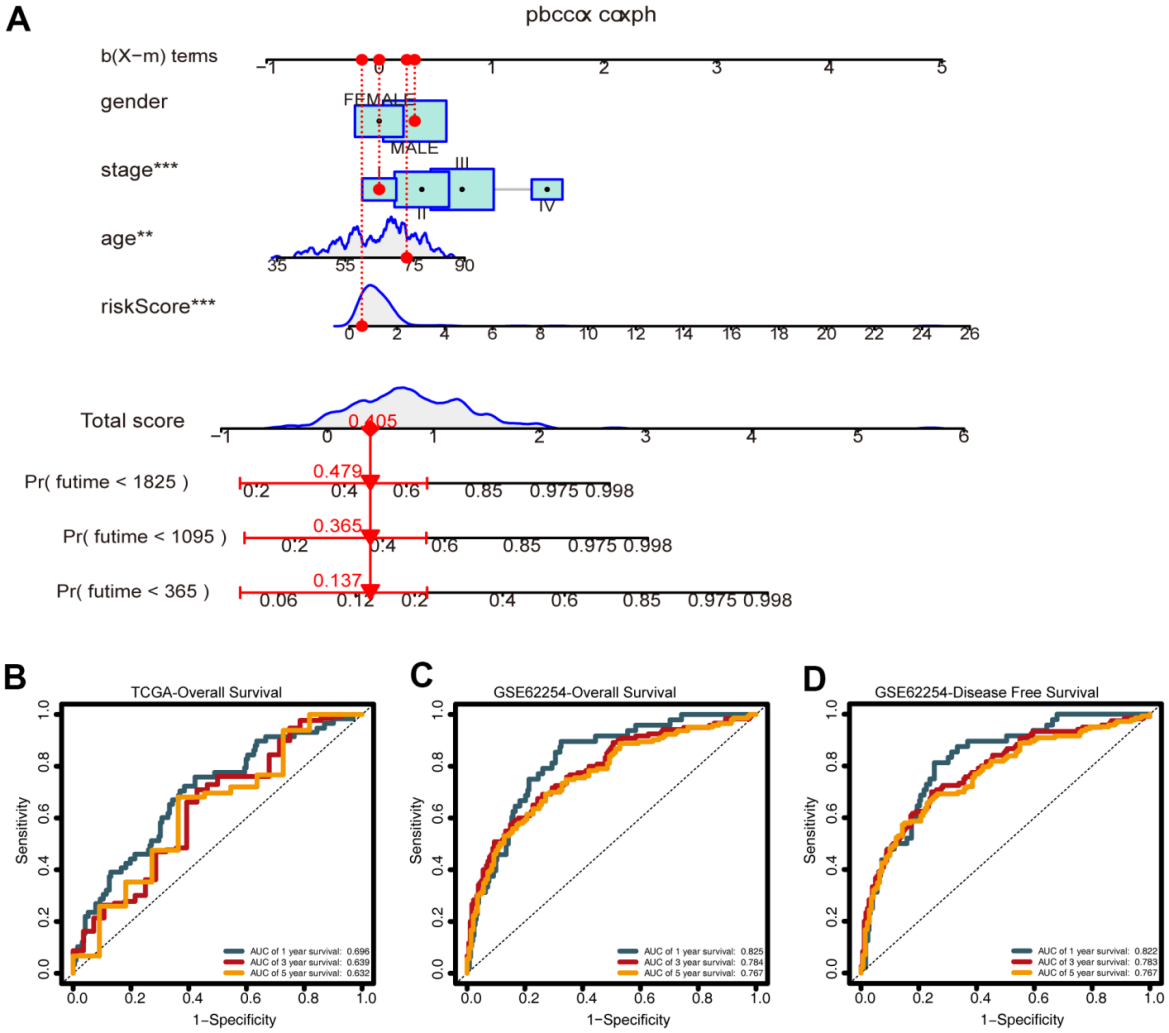
**Construction of the nomogram.** (**A**) A nomogram for predicting one-, three-, and five-year overall survival (OS) generated by integrating the risk score, age, gender, and stage. (**B**) Time-dependent receiver operating characteristic ROC curves of the nomogram for OS prediction from the training set. (**C**) Time-dependent ROC curves of the nomogram for OS prediction from the validation set. (**D**) Time-dependent ROC curves of the nomogram for DFS predictions from the validation set.

To predict the survival of GC patients, we constructed a nomogram based on the training set, which included the CDG signature risk score, age, gender, and pathological stage (Figure 8A). Time-dependent ROC analysis was performed to assess the predictive accuracy of the nomogram. Plotting the one-, three-, and five-year ROC values of OS in the training set of the nomogram revealed AUC values of 0.696, 0.639, and 0.632, respectively (Figure 8B). Time-dependent ROC analyses from the nomogram for one-, three- and five-year OS probabilities in the validation set returned AUC values of 0.825, 0.784, and 0.767, respectively (Figure 8C). In addition, ROC analysis of the DFS predictions in the validation set revealed that the nomogram was highly discriminatory, with AUC values of 0.825, 0.784, and 0.767 for one-, three-, and five-year DFS levels, respectively (Figure 8D).

Calibration and decision curve analysis (DCA) revealed the reliability of the nomogram for predicting prognoses in the training and validation sets. The calibration plot revealed that the predictions made using the nomogram were consistent with the true observations (Supplementary Figure 2A–2C). The DCA curves for the predictive nomogram revealed that it had high net benefit (Supplementary Figure 2D–2F). The web-based calculator (https://prognosis.shinyapps.io/STAD/) could predict the OS of GC patients based on the established nomogram (Supplementary Figure 3A, 3B) and is convenient in terms of its usage and visualization of the prognostic nomogram.

## DISCUSSION

Prognostic outcomes of GC patients are highly variable. Thus, there is an urgent need to find new GC biomarkers and to construct new prognostic models for predicting GC survival in order to develop personalized treatment plans [9]. In this study, we developed a prognostic CDG signature and a corresponding nomogram for predicting GC patient survival. We have developed a promising tool for predicting GC outcomes and guiding personalized GC therapy.

Altering the expression of CDGs can increase cell proliferation and survival, leading to clonal expansion and tumor growth [10]. Different cancer types may be associated with both common and specific driver genes, and different genes may play different roles in various cancer types. Here, we developed a seven-CDG prognostic signature based on *DDB2*, *ENPEP*, *GNAS*, *MSI2*, *MYO5A*, *PLAG1*, and *RBM15*. DDB2 was originally identified as a novel tumor suppressor via nucleotide excision repair [11], and it is abnormally expressed in several tumor tissues [12–15]. However, increasing evidence suggests that DDB2 exhibits dual functions in cancer cell proliferation. Qiao et al. reported that DDB2-silencing inhibits proliferation and migration of GC cells [16]. ENPEP is an essential and highly specific proangiogenic enzyme. ENPEP functions in tumor proliferation, migration, and drug resistance in breast and colorectal cancers [17, 18]. *GNAS* is a complex gene locus that gives rise to multiple translated and non-translated gene products [19–22]. At present, few studies have investigated the role of *GNAS* in GC, thus future studies should systematically elucidate its functions. MSI2 is a member of the Musashi family of RNA-binding proteins, which are overexpressed in various tumors, including ovarian, pancreatic, bladder, and lung cancers [23–26]. MSI2 overexpression is correlated with poor prognoses of liver and pancreatic cancer patients [27, 28]. Early studies on MYO5A focused on its roles in neuron formation and function, and in neurological disease. However, the functions and clinical significance of MYO5A in GC remain unclear. Recent studies have reported that MYO5A plays a role in tumorigenesis. Zhao et al. reported that serum MYO5A levels are a valuable predictor of cervical nodal occult metastasis and can be used to assess prognosis [29]. PLAG1 is a transcription factor involved in various cancers, such as lipoblastoma, hepatoblastoma, acute myeloid leukemia, uterine leiomyoma, and leiomyosarcoma [30]. RBM15, which is a member of the split ends family of proteins, determines cell-fate in many tissues including blood and is overexpressed in hepatocellular carcinoma [31]. While the studies highlighted above have revealed the functions of these CDGs in other cancers, few studies have investigated the roles of these CDGs in GC tumorigenesis.

While several studies have focused on the functions of CDGs, systematic analysis of their prognostic potential for GC is still required. The CDG signature identified in our study was significantly associated with the survival of GC patients, and this association remained significant after controlling for clinical and pathological features. We constructed a nomogram for predicting one-, three- and five-year OS values for GC based on this CDG signature, age, gender, and stage. ROC analysis, calibration plots, and DCA were used to verify the prognostic accuracy of the model, and the results showed that this model had strong predictive ability. We also created a simple online tool to perform this analysis in clinical settings.

To further our understanding of the mechanisms associated with this CDG signature, GSEA was conducted to compare the low- and high-risk groups. Terms and categories such as “calcium signaling pathway,” “CAMs,” “extracellular matrix receptor interaction,” “focal adhesion,” “gap junction,” “collagen-containing extracellular matrix,” “contractile fiber,” “glycosaminoglycan binding,” “hormone binding,” and “muscle system processes” were highly enriched in the high-risk group, indicating that the seven CDGs are involved in these signaling pathways in GC.

There is evidence that CDGs are closely related to tumor cell immune infiltration. In non-small cell lung cancer, GNAS promotes migration and invasion of cancer cells by altering macrophage polarization [32]. However, studies focusing on the role of the seven CDGs, identified in this study, in immune infiltration remain limited. In the present study, CIBERSORT was used to calculate the proportions of 22 immune cell subsets in GC, revealing that the high-risk group had high proportions of activated NK cells, monocytes, M2 macrophages, resting dendritic cells, and resting mast cells. These findings provide insight into the mechanisms associated with these CDGs in GC.

Many previous studies have constructed prognostic gene signatures. Chen et al. constructed a stemness index-related signature for GC with an AUC value of 0.688 [33]. Ren et al. reported angiogenesis-related gene expression signatures for predicting DFS in GC patients with an AUC value of 0.673 [34]. ROC analysis in the validation group for the nomogram in our study (0.825, 0.784, and 0.767 for OS and 0.822, 0.783, and 0.767 for DFS) indicated that the prognostic index is a stable predictor for the prognosis of GC patients. We also performed ROC analysis with traditional clinicopathological predictors (stag and grade), which demonstrated that our CDG signature has high prognostic value compared to that of these predictors.

However, our study has the following limitations. First, the data used in this study were obtained from two different databases and a non-database case was not used for external verification. Second, we did not investigate the mechanisms underlying these CDGs in GC. Third, the levels of immune cell infiltration were calculated based on algorithmic evaluations and, thus, require experimental validation. Therefore, further genetic and experimental studies with larger sample sizes and experimental validation are needed.

In summary, this is the first study to identify and validate a CDG signature that could independently predict the OS and DFS of GC patients. A prognostic nomogram was constructed by integrating age, sex, and TNM stage, which performed well in predicting the survival of GC patients. Our study, thus, generated a clinically useful tool for improving prognostic management of GC.

## MATERIALS AND METHODS

### Data collection and processing

Publicly available transcriptomic and clinical data associated with GC samples were obtained from TCGA (https://tcga-data.nci.nih.gov/tcga/) and the Gene Expression Omnibus (GEO; https://www.ncbi.nlm.nih.gov/gds/) and analyzed retrospectively. We used the RNA-Seq fragments per kilobase of transcript per million mapped reads (FPKM) data from TCGA. After excluding cases with follow-up times of <30 days, 634 patients were enrolled in the study, including 334 patients from TCGA-STAD project and 300 from the GSE62254 cohort [35]. Data from the GSE62254 cohort were obtained from the Asian Cancer Research Group (ACRG) Gastric cohort, which included 199 male and 91 female GC patients. The median age was 64 years and the range was 24-86 years.

Data for 568 CDGs (Supplementary Table 1) were downloaded from the Integrative OncoGenomics (IntOGen) pipeline (https://www.intogen.org/search) [7]. The immunohistochemical data associated with proteins encoded by each CDG in GC and normal tissues were obtained from the HPA (https://www.proteinatlas.org/).

### Construction of the CDG signature

To narrow down the screening range, overlapping prognostic CDGs were selected from TCGA-STAD and GSE62254 cohorts via Cox univariate analysis. TCGA-STAD and GSE62254 were then used for model training (*n* = 334) and validation (*n* = 300), respectively. Previously selected CDGs were further screened and confirmed by LASSO Cox regression analysis (with the penalty parameter estimated by 10-fold cross-validation) using the “glmnet” package. A formula was developed using the CDG signature constructed above, where β corresponds to the correlation coefficient:

Risk score = β1 × (expression of RNA1) + β2 × (expression of RNA2) + ··· + βn × (expression of RNA*n*)

The patients in each dataset were assigned to a high- or a low-risk group using the median risk score as a cutoff. The ROC curves were created using the “survivalROC” package, and the AUC values were calculated to evaluate the predictive potential of the CDG signature.

### Validation of the CDG signature

To validate the CDG signature, the patients in the validation set were separated into high- or low-risk groups according to the median risk score, which was calculated according to the CDG signature. Kaplan-Meier curve and time-dependent ROC analyses were conducted to assess CDG signature categorization.

### Subgroup analysis of the CDG signature

To validate the effectiveness of the prognostic CDG signature, stratification analysis was performed on the training and validation sets using different demographic and clinical characteristics. The GC cases were divided into two risk groups according to their characteristics and risk scores, and Cox regression analysis was performed to analyze differences between the subgroups.

### Estimation of immune cell infiltration

To analyze the relationship between the CDG signature and immune cell characteristics, CIBERSORT was used to estimate the fractions of immune cell types between the high- and low-risk groups [36]. Statistical analysis of the proportions of 22 immune cell types in each of the 334 GC samples was performed using the Wilcoxon rank-sum test.

### GSEA

GSEA was performed to explore the GO terms and KEGG pathways that were significantly enriched in high-risk GC samples (http://www.broadinstitute.org/gsea). Gene sets were considered significantly enriched when FDR < 0.05 and |NES| > 1.

### Construction and evaluation of the nomogram

We designed a novel nomogram model containing the CDG signature and clinicopathological predictors to establish a quantitative clinical tool to monitor and predict outcomes of GC patients. Subsequently, we developed a web-based calculator based on this model for clinical applications. Time-ROC curves and calibration plots were generated, and DCA was performed to evaluate the clinical utility of the novel nomogram.

### Statistical analysis

All statistical analyses were performed using R software (version 4.0.5, R Development Core Team, 2021) and GraphPad Prism (version 8.3.0, GraphPad software, Inc., San Diego, CA, USA). All statistical tests (two-tailed) with *P* < 0.05 were considered statistically significant.

## Supplementary Material

Supplementary Figures

Supplementary Table 1
