# Supplementary material for: Establishing a cancer driver gene signature-based risk model for predicting the prognoses of gastric cancer patients
Source: Aging (Albany NY). 2022 Mar 14;14(5):2383–99. doi: 10.18632/aging.203948 (PMC8954960; doi:10.18632/aging.203948)
Supplement: Supplementary Figures [file aging-14-203948-s001.pdf]

SUPPLEMENTARY FIGURES

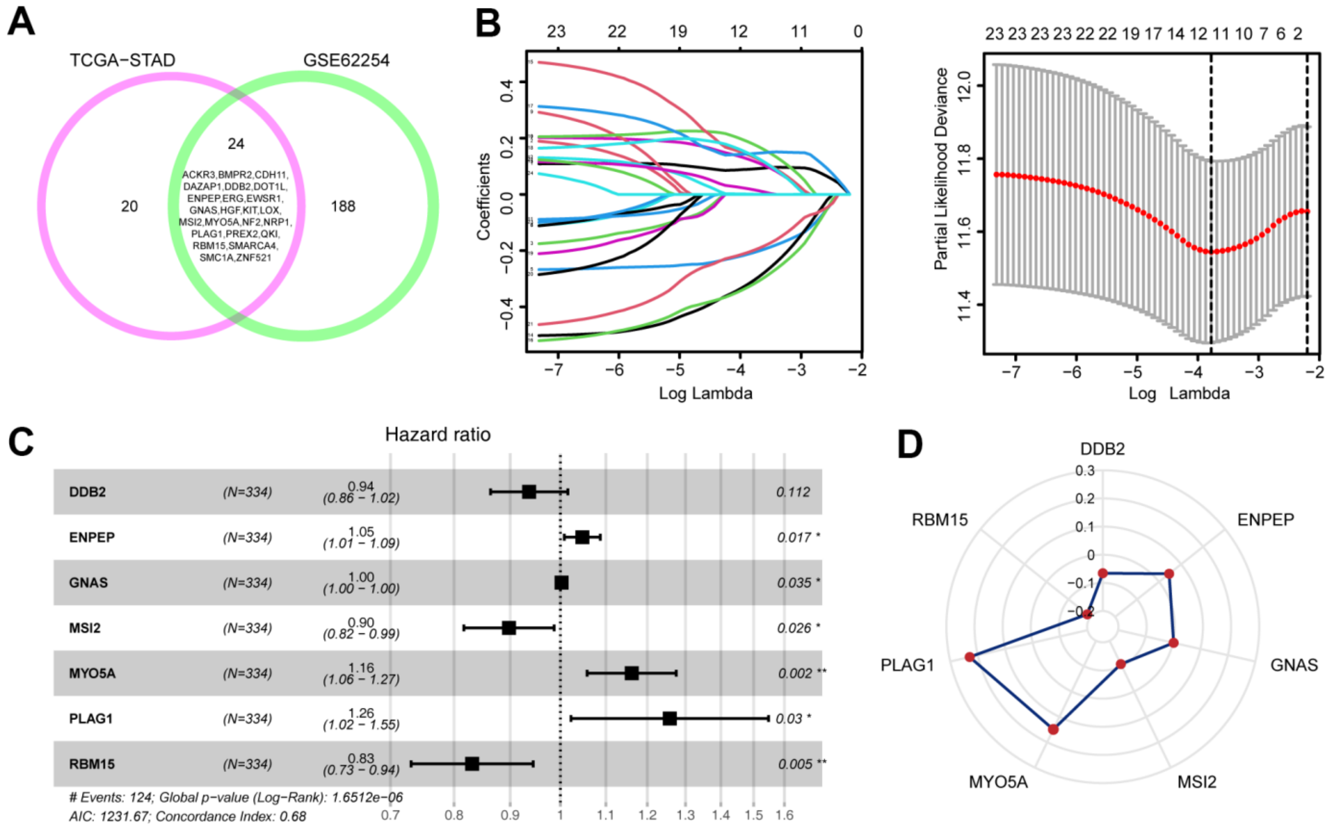

Supplementary Figure 1. Establishment of a prognostic CDG (cancer driver gene) signature. (**A**) Screening of prognosis-related CDGs. (**B**) Least absolute shrinkage and selection operator (LASSO) regression analysis to screen prognosis-related genes from the survival-related CDGs. (**C**) Forest plot of multivariate Cox regression analyses used to construct the prognostic CDG signature. (**D**) Seven-CDG signature for GC based on Cox regression coefficient.

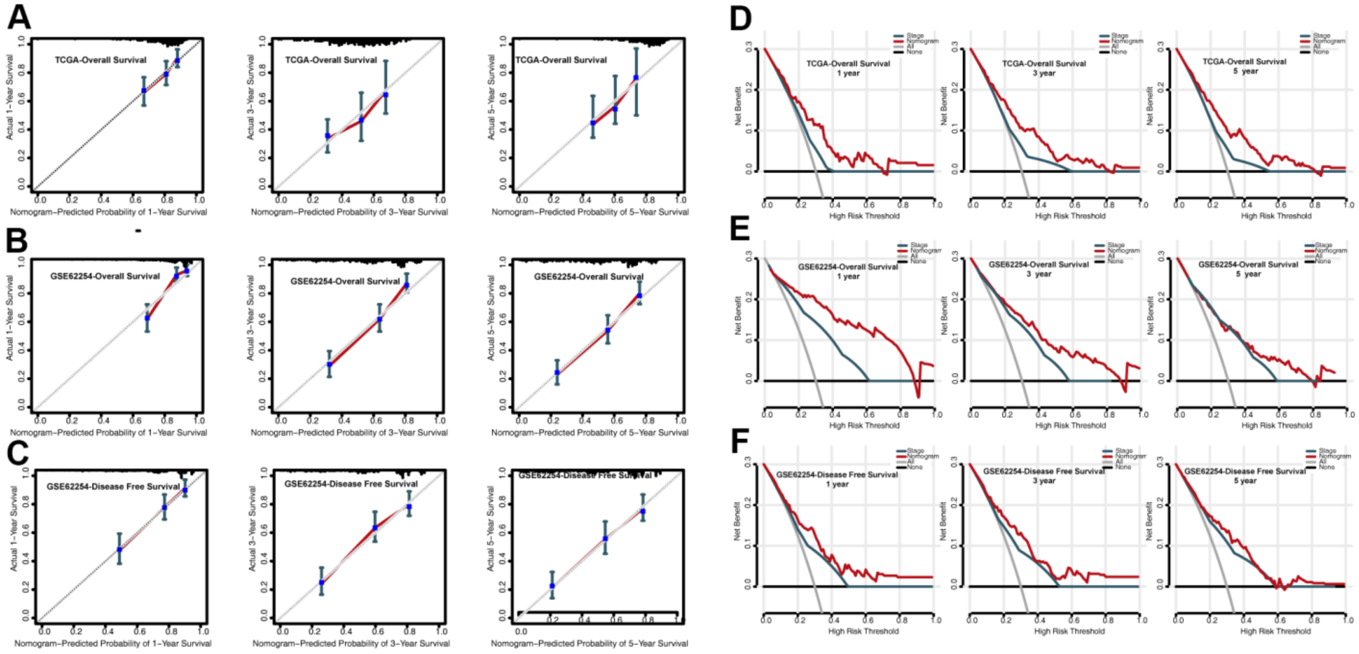

Supplementary Figure 2. Evaluation of the cancer driver gene (CDG)-based nomogram. (**A**) Calibration plot for overall survival (OS) prediction from the training set of the nomogram. (**B**) Calibration plot for OS prediction from the validation set of the nomogram. (**C**) Calibration plot for disease-free survival (DFS) prediction from the validation set of the nomogram. (**D**) Decision curve analysis (DCA) for OS prediction from the training set of the nomogram. (**E**) DCA for OS prediction from the validation set of the nomogram. (**F**) DCA for DFS prediction from the validation set of the nomogram.

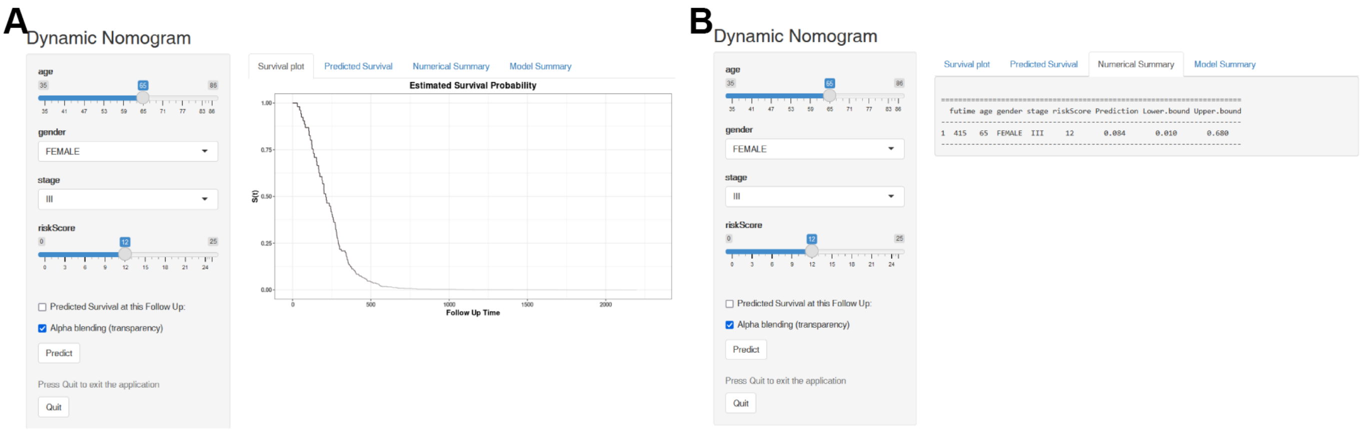

Supplementary Figure 3. Establishment of an easy-to-operate web-based calculator for predicting gastric cancer (GC) prognoses (https://prognosis.shinyapps.io/STAD/). (**A**) Overall survival rate calculator. (**B**) 95% confidence interval of the overall survival rate determined using the web-based calculator.
